# Uncovering Harmonization Potential in Health Care Data Through Iterative Refinement of Fast Healthcare Interoperability Resources Profiles Based on Retrospective Discrepancy Analysis: Case Study

**DOI:** 10.2196/57005

**Published:** 2024-07-23

**Authors:** Lorenz Rosenau, Paul Behrend, Joshua Wiedekopf, Julian Gruendner, Josef Ingenerf

**Affiliations:** 1 IT Center for Clinical Research University of Lübeck Lübeck Germany; 2 Chair for Medical Informatics Friedrich-Alexander-Universität Erlangen-Nürnberg Erlangen Germany; 3 Institute of Medical Informatics University of Lübeck Lübeck Germany

**Keywords:** Health Level 7 Fast Healthcare Interoperability Resources, HL7 FHIR, FHIR profiles, interoperability, data harmonization, discrepancy analysis, data quality, cross-institutional data exchange, Medical Informatics Initiative, federated data access challenges

## Abstract

**Background:**

Cross-institutional interoperability between health care providers remains a recurring challenge worldwide. The German Medical Informatics Initiative, a collaboration of 37 university hospitals in Germany, aims to enable interoperability between partner sites by defining Fast Healthcare Interoperability Resources (FHIR) profiles for the cross-institutional exchange of health care data, the Core Data Set (CDS). The current CDS and its extension modules define elements representing patients’ health care records. All university hospitals in Germany have made significant progress in providing routine data in a standardized format based on the CDS. In addition, the central research platform for health, the German Portal for Medical Research Data feasibility tool, allows medical researchers to query the available CDS data items across many participating hospitals.

**Objective:**

In this study, we aimed to evaluate a novel approach of combining the current top-down generated FHIR profiles with the bottom-up generated knowledge gained by the analysis of respective instance data. This allowed us to derive options for iteratively refining FHIR profiles using the information obtained from a discrepancy analysis.

**Methods:**

We developed an FHIR validation pipeline and opted to derive more restrictive profiles from the original CDS profiles. This decision was driven by the need to align more closely with the specific assumptions and requirements of the central feasibility platform’s search ontology. While the original CDS profiles offer a generic framework adaptable for a broad spectrum of medical informatics use cases, they lack the specificity to model the nuanced criteria essential for medical researchers. A key example of this is the necessity to represent specific laboratory codings and values interdependencies accurately. The validation results allow us to identify discrepancies between the instance data at the clinical sites and the profiles specified by the feasibility platform and addressed in the future.

**Results:**

A total of 20 university hospitals participated in this study. Historical factors, lack of harmonization, a wide range of source systems, and case sensitivity of coding are some of the causes for the discrepancies identified. While in our case study, Conditions, Procedures, and Medications have a high degree of uniformity in the coding of instance data due to legislative requirements for billing in Germany, we found that laboratory values pose a significant data harmonization challenge due to their interdependency between coding and value.

**Conclusions:**

While the CDS achieves interoperability, different challenges for federated data access arise, requiring more specificity in the profiles to make assumptions on the instance data. We further argue that further harmonization of the instance data can significantly lower required retrospective harmonization efforts. We recognize that discrepancies cannot be resolved solely at the clinical site; therefore, our findings have a wide range of implications and will require action on multiple levels and by various stakeholders.

## Introduction

### Overview

Interoperability, an essential component of contemporary medical informatics, facilitates seamless communication and data exchange between various devices, applications, and health care systems. Semantic interoperability ensures machine interpretation of health care data and, thus, data exchange, integration, and reuse for optimized collaboration between distributed players in health care and medical research. Consequently, it is pivotal in amplifying the efficacy and impact of numerous other medical informatics technologies, advancing the field as a whole.

### The Layered Fast Healthcare Interoperability Resources Profile Model: Facilitating Reuse and Interoperability

The Health Level 7 (HL7) Fast Healthcare Interoperability Resources (FHIR) addresses syntactic and semantic interoperability [1], providing a standardized framework for data structures known as resources. These resources are essentially a set of attributes that represent specific health care–related concepts. For instance, the *Patient* resource in FHIR might include attributes such as name, administrative gender, birth date, address, and contact details. This standardization of data structure promotes syntactic interoperability. Semantic interoperability is achieved using bindings of attributes to value sets with codes from universally recognized coding systems such as *Logical Observation Identifiers Names and Codes* (LOINC) or *Systematized Nomenclature of Medicine—Clinical Terms* (SNOMED CT).

FHIR also introduces the concept of *Profiling* [2]. FHIR profiles constrain or modify the base FHIR resources to cater to specific use cases or regional requirements. They provide guidelines on how resources should be structured; which attributes should be excluded, mandatory, added, or repeated; what terminology should be used for coded elements; and how these elements should be interpreted. During the development of profiles, domain experts from the medical field are involved at all stages to ensure that the data models capture all the required data. This approach ensures the syntactic consistency of the data and its semantic interpretability. FHIR profiles can be based on other FHIR profiles to constrain them further, allowing a layered structure from the most permissive to the most constrained profile.

### The Medical Informatics Initiative

The German government recognized the importance of interoperability in the health care sector and consequently initiated the Medical Informatics Initiative (MII) in 2015 [3]. With the overarching goal of making routine data available for research, the MII aims to digitally connect patient data generated during hospital stays across the country. Four consortia, namely, DIFUTURE [4], HiGHmed [5], Medical Informatics in Research and Care in University Medicine [MIRACUM] [6], and Smart Medical Information Technology for Health care [7], and a central coordination office received funding from the Federal Ministry of Education and Research to establish data integration centers (DICs) responsible for data exchange. With over €400 (US $427 million) million in current total funding, the Federal Ministry of Education and Research supports consortia, DICs, and cross-consortium use cases.

Acknowledging the various accomplishments within the MII until this point [8-10], in this paper, we will place particular emphasis on the Core Data Set (CDS), its implementation at the DICs, and its applicability to the “Aligning Biobanking and DIC Efficiently” [11] use case project, which among others, included federated feasibility studies integrated into the *Forschungsdatenportal für Gesundheit* (German Portal for Medical Research Data [FDPG]) [12].

### CDS Profiles

One of the primary responsibilities of the MII is the development and implementation of a unified data model that is binding for all German university hospitals.

The result of this ongoing endeavor within the MII is the CDS, a set of specific FHIR profiles that the local sites have agreed upon collectively. These CDS profiles define the minimum data set that should be included in each DIC. The CDS is subdivided into basic and extension modules, where the basic modules encompass basic health care data such as patient-derived information, conditions, procedures, medication, and laboratory measures, and the extension modules reflect data from specific applications or specialist areas (such as intensive care or oncology) [13]. The CDS is sustainably, nationally coordinated, continuously updated, and adapted to meet changing requirements. The development of the CDS leverages tools such as ART-DECOR for data set modeling and Simplifier.net [14] for creating and publishing FHIR profiles [15].

Upon the successful development of the CDS, the obligation falls to each DIC to make its routine data available as FHIR instance data. DICs integrate and standardize routine health care data at each site, essentially based on extract, transfer, load (ETL) processes with frequent late mappings of proprietary data from source systems. While their operation and management involve complex processes, for the scope of this paper, this conceptualization is sufficient. The data are secured and standardized through the DIC, promoting efficient and secure cross-institutional data sharing and collaboration.

### FDPG: Facilitating Accessible Research

The FDPG is critical in making the data across the 34 sites accessible to medical researchers [12]. It provides the legal and procedural framework to access routine data sets across sites. Among other components, it provides the feasibility platform that gives users a user-friendly view of available data items and allows users to query them across connected sites directly. It aggregates available patient counts for a specific, user-defined search query. To do so, it uses a search ontology automatically generated from FHIR profiles [16]. Users can select concepts from the search ontology and restrict them as needed, for example, by applying comparator values for quantitative laboratory values. The resulting criteria can be combined using Boolean algebra to create complex feasibility queries.

**Figure 1:**
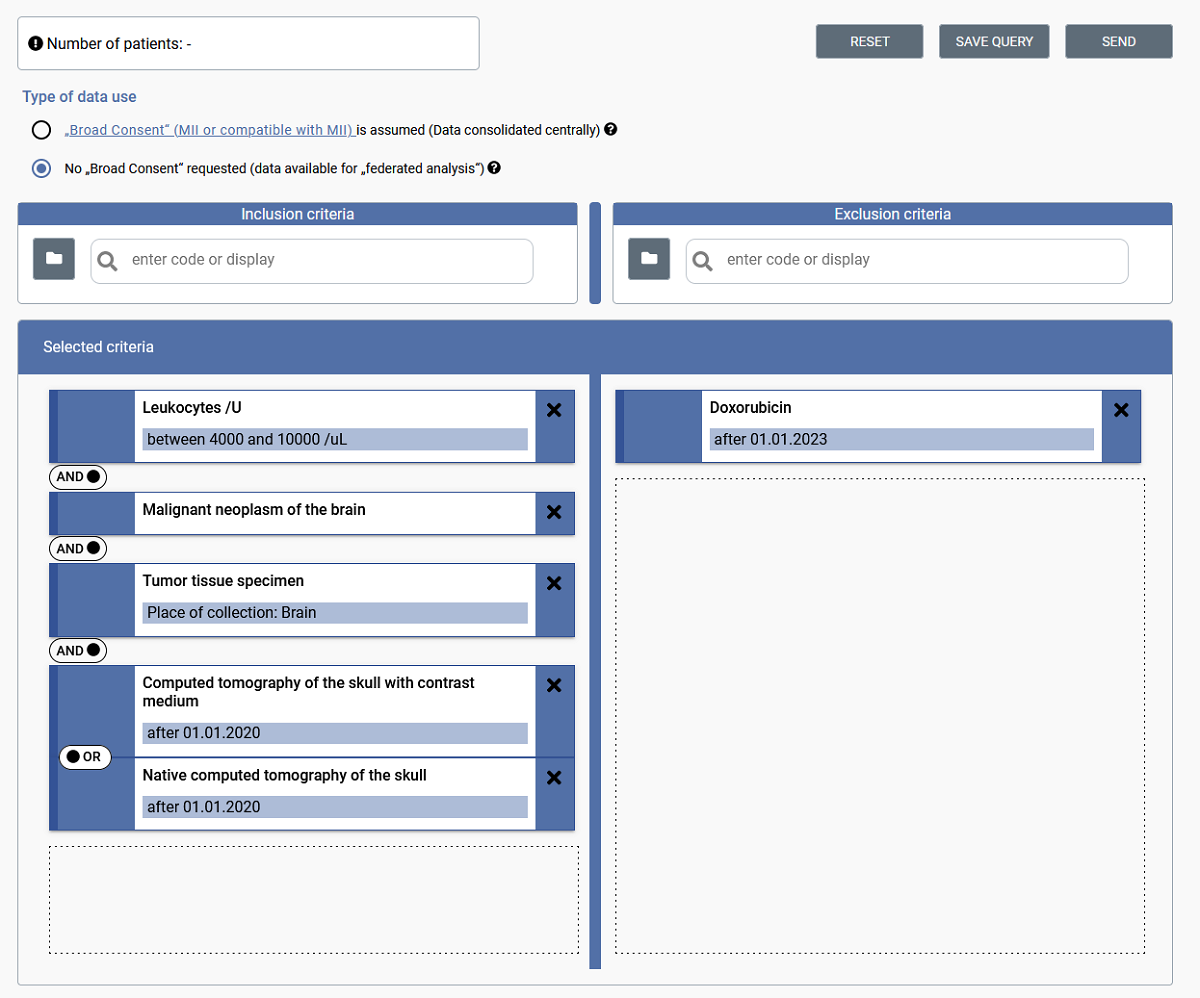
Example of a feasibility query in the German Portal for Medical Research Data feasibility portal to find patients with a leukocyte count within a normal range, those with a malignant neoplasm of the brain, those with available tumor tissue specimen, and those with a computed tomography scan after January 1, 2020, who did not take doxorubicin after January 1, 2023. MII: Medical Informatics Initiative.

Figure 1 demonstrates the search ontology’s user-friendly abstraction for researchers unfamiliar with FHIR:

- *Medical coding:* criteria are based on standard codings referenced in the FHIR profiles (eg, C71 from *German modification of the International Statistical Classification of Diseases and Related Health Problems, 10th revision* [*ICD-10-GM*] for malignant neoplasm of the brain)
- *Value and attribute filters:* specific FHIR attributes such as the value of an observation are modeled as “value filters” to express the “is” relationship between the coding and the value (such as leukocyte counts between 4000 and 10,000/µL). Additional attributes can also be expressed (such as place of collection for specimen) and allow the further refinement of criteria beyond their existence.
- *Time-based filters:* researchers can furthermore apply temporal constraints (“after” a specific date).

All other FHIR attributes are not available to the user to ensure high usability.

The FDPG feasibility tool’s primary aim is to make the data findable that are available across the MII’s DICs, which are based on the CDS. In its current iteration, the feasibility tool offers a selected subset of CDS criteria. This design choice is driven by the different requirements to be met by the CDS and the search ontology. The CDS profiles are developed for various primary source systems across German university hospitals. Profile specifications are required to be broad in many cases due to the diversity and complexity of these systems. Therefore, generic profiles are preferred, with more detailed models being developed only when necessary for specific content or organizational reasons. This serves DICs well due to having access to the instance data. By contrast, due to its federated nature, the FDPG has no access to the instance data. A more nuanced approach beyond generic modulations, such as accurately modeling the relationship between laboratory concepts and their values instead of simply representing LOINC codes, is required to provide users with criteria beyond determining their presence.

The feasibility tool also navigates the complexity arising from various code systems. For instance, the diagnosis profile accommodates codings from *ICD-10-GM*, SNOMED CT, Alpha-ID, and Orphanet, which are crucial for rare disease research. However, this diversity poses a usability challenge, potentially overwhelming users unfamiliar with these systems. In addition, the overlap in concept expression between *ICD-10-GM* and SNOMED CT can create confusion: users may not realize the necessity of selecting a particular concept from a specific code system or potentially both, contingent on their use case. A significant factor in the FDPG feasibility portal’s initial focus on legally mandated code systems is the absence of instance data for certain criteria (eg, as of March 2023, none of the 20 participating sites had SNOMED CT codings for diagnoses), as offering such criteria might frustrate researchers and discourage tool use. Guided by these assumptions, Table 1 provides an overview of the CDS modules, their codings, and their coverage in the FDPG feasibility portal.

The present scope of the FDPG feasibility portal is not fixed, allowing for potential future expansions.

**Table 1:** Coverage of the Core Data Set (CDS) modules in the German Portal for Medical Research Data (FDPG) feasibility portal.

CDS module	CDS-supported codings	FDPG coverage
Consent	MII^a^_CS_Consent_Policy	MII_CS_Consent_Policy
Diagnosis	*ICD-10-GM*^b^, Alpha-ID, SNOMED^c^ diagnoses codes, Orphanet	*ICD-10-GM*
Laboratory	LOINC^d^	Defined subset of “TOP300” LOINC codes
Medication	ATC^e^-DE, ATC-EN, PZN^f^	ATC-DE
Person	—^g^	—
Procedure	OPS^h^, SNOMED procedure codes	OPS
Specimen	SNOMED specimen codes	Defined subset of “TOP50” SNOMED specimen codes

^a^MII: Medical Informatics Initiative.

^b^*ICD-10-GM*: *German modification of the International Statistical Classification of Diseases and Related Health Problems, 10th revision*.

^c^SNOMED: Systematized Nomenclature of Medicine.

^d^LOINC: Logical Observation Identifiers Names and Codes.

^e^ATC: Anatomical Therapeutic Chemical.

^f^PZN: pharmazentralnummer.

^g^Not applicable.

^h^OPS: Operationen- und Prozedurenschlüssel.

### Discrepancy Despite Standardization: Challenges and Opportunities

**Figure 2:**
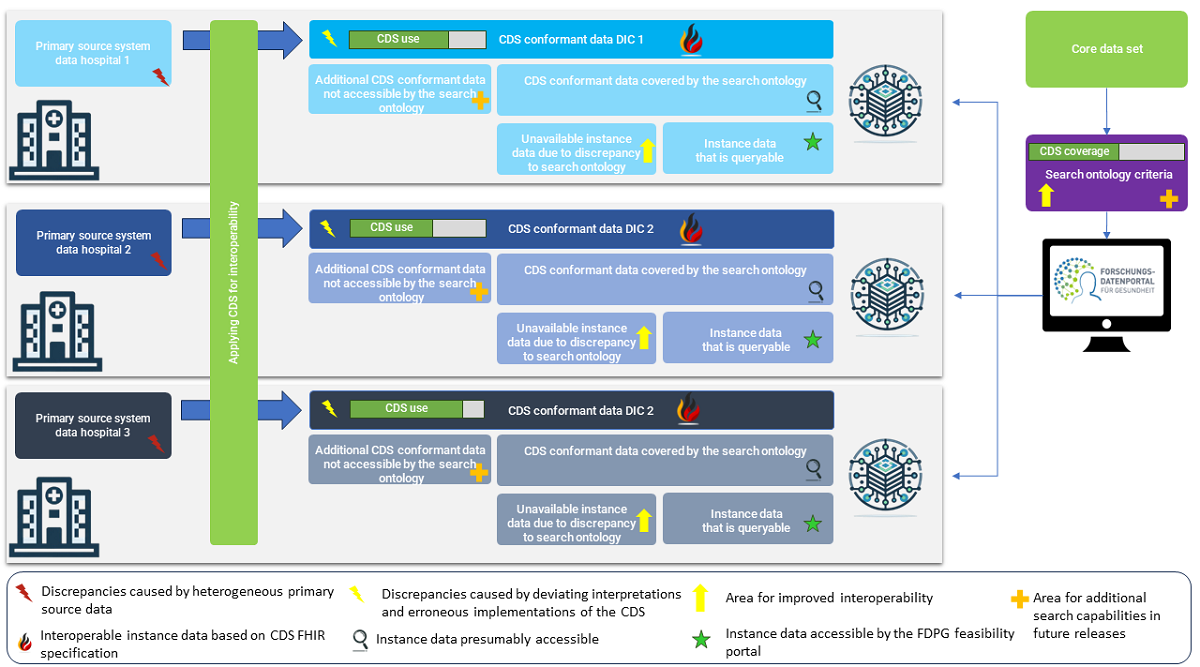
Simplified federated architecture of the German Portal for Medical Research Data and the data integration center and the data discrepancies and alignments that arise. CDS: Core Data Set; DIC: data integration center; FHIR: Fast Healthcare Interoperability Resources.

Figure 2 illustrates the interplay between the source systems, the DICs, the CDS, and the search ontology of the FDPG feasibility portal. Applying the CDS to the heterogeneous primary source data makes the heterogeneous source data interoperable. Despite the standardization, discrepancies can arise by deviating interpretations or erroneous implementations of the CDS. Typically, these can be identified by validating the instance data against the CDS and are addressed in the ETL jobs of the sites. It might be necessary to adjust the CDS implementation guide to provide more clarity. It is important to note that despite these efforts, inherent challenges related to data quality at the source, such as missing, erroneous, or inconsistently entered data, persist [17]. These complexities often necessitate extensive collaboration and resources for resolution and fall outside the direct control of DICs, whose primary function is data integration.

The FDPG feasibility portal cannot exhaustively query all instance data at the clinical sites. Consequently, the instance data can be divided into 2 subsets: the data that should be covered by the search ontology and the currently unsupported data. The latter provides opportunities for future developments but is outside the scope of this paper.

The search ontology’s additional constraints and the CDS’s limited data harmonization cause discrepancies that prevent the accessibility to instance data that should be available. While presenting challenges, such discrepancies are not unique to this framework but are commonly observed across various industries when implementing standards [18]. They mirror the common rationale behind the organization of Connectathons, which aim to test and improve interoperability.

Outside the governmental context of a Connectathon but maintaining the same objective of advancing interoperability through rigorous testing of multiple systems adhering to the same standard, our approach evaluates the assumptions underlying the FDPG’s search ontology against the actual instance data at clinical sites. To achieve this, we use the same FHIR profiles used to develop the search ontology.

## Methods

### Overview

**Figure 3:**
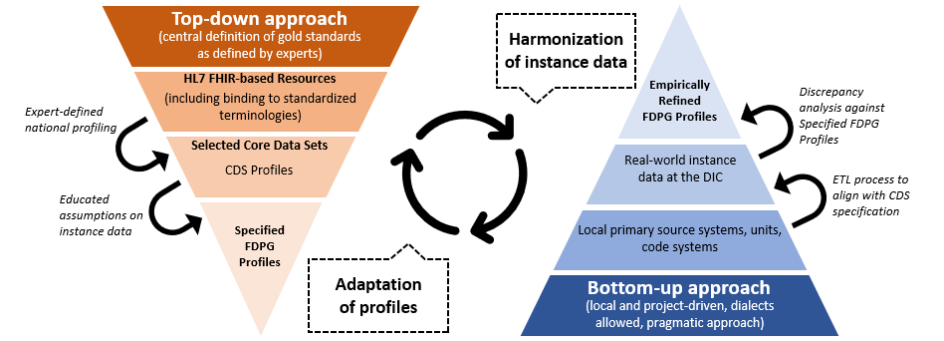
Combining the top-down and bottom-up approach for the iterative, evidence-based refinement of FHIR profiles. CDS: Core Data Set; DIC: data integration center; ETL: extract, transfer, load; FDPG: German Portal for Medical Research Data; HL7 FHIR: Health Level Seven Fast Healthcare Interoperability Resources.

This research integrates a top-down approach for the definition of data models with an empirical bottom-up methodology (Figure 3). This novel approach addresses discrepancies in data standardization and representation. The representation of the search ontology as an FHIR profile based on the CDS forms the foundation of our top-down perspective (hereinafter referred to as FDPG profiles). The bottom-up approach, conversely, is grounded in an empirical analysis of the instance data.

Being able to rely on FHIR profiles has multiple advantages:

- *Using existing validation software:* unlike custom solutions, our approach leverages existing validation software to analyze and resolve discrepancies, offering a more streamlined and efficient process.
- *Insights from layered FHIR profiles:* the layered structure of FHIR profiles provides multifaceted insights. It not only aids in understanding the search ontology but also evaluates compliance with the CDS. In addition, performing this analysis across different sites sheds light on several aspects: (1) discrepancies in instance data across sites, (2) discrepancies between the CDS and site-specific instance data, and (3) discrepancies between the search ontology and the instance data
- *Leveraging established standards:* working with established technology in the MII allows for transparency and adaptability beyond the current use case.

The strength of our approach lies in its iterative nature, whereby each cycle involves refining the profiles based on the empirical insights gathered, subsequently informing the following empirical analysis. This continuous refinement allows us to transition from differential to data quality analysis. This progression not only targets the resolution of discrepancies but also seeks to enhance the quality and accessibility of the data. By iterating this process, we contribute a new framework for reconciling the tension between data standardization and real-world variability, a common challenge in health care data management. Notably, the approach is not solely focused on the search ontology, as would typically be the case when developing a product. Being a part of the MII community, we hope that our approach will also uncover potential for further harmonization across sites, enhancing the functionality and accessibility of the FDPG feasibility tool and facilitating a wider range of use cases.

### Validation Pipeline

**Figure 4:**
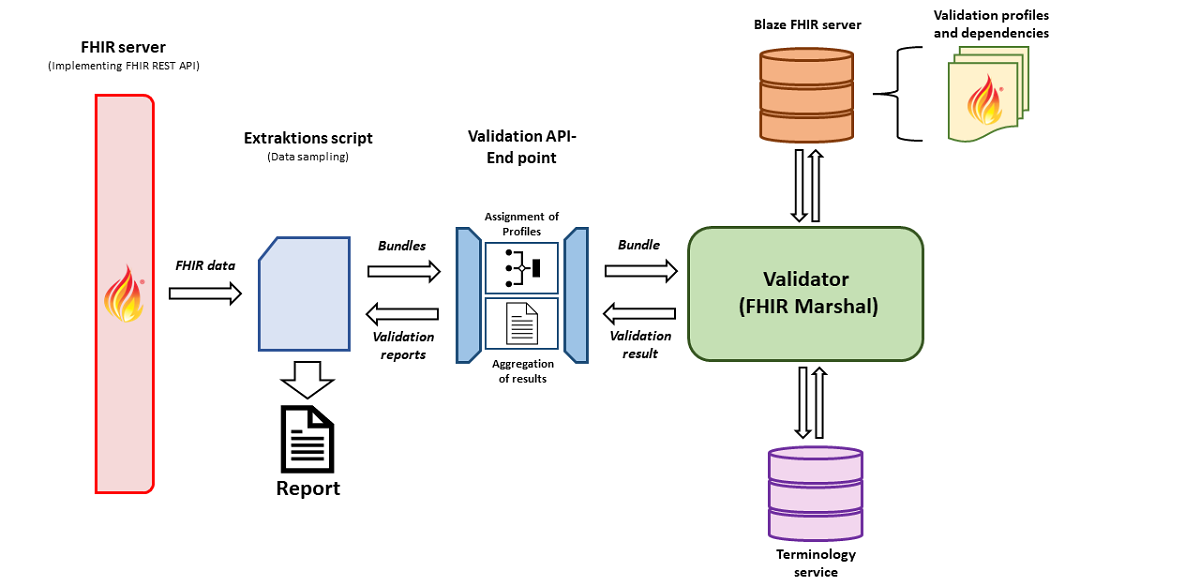
Validation pipeline. API: application programming interface; FHIR: Fast Healthcare Interoperability Resources; REST: Representational State Transfer.

To use the FDPG profiles, we developed and deployed a validation pipeline (Figure 4) that can be deployed at each site. A centralized approach was not feasible due to the high standards of data protection adhered to within the MII. The pipeline uses the FHIR Marshal, an HL7 application programming interface validation library extended with a Representational State Transfer application programming interface [19]. The validation library requires the profiles and their dependencies for the validation as well as the referenced terminology resources.

The FHIR profiles are themselves resources, more specifically, FHIR StructureDefinition resources. They are stored in an FHIR server (Blaze) [20] using the *FHIR Populator* tool [21]. This tool is designed to download FHIR profiles from package managers such as Simplifier and their dependencies, subsequently uploading them to our servers. The FHIR Populator also accommodates environments without internet access by offering the capability to upload a previously persisted package.

Regarding terminologies, we analyzed all StructureDefinitions and downloaded the expanded ValueSets from a terminology server based on the binding information. The terminologies are stored in a specialized FHIR server, Termite [22], which implements a minimal set of FHIR Terminology Services for validation and contains all relevant expanded ValueSets and CodeSystems [23].

The process chain uses a Python script to extract equivalence class test samples (for each profile, a maximum of 500 instances) from the local FHIR server. Within the meta information of each resource, the profile it implements is listed. By substituting this profile identifier from the CDS with the corresponding FDPG profile identifier, we can use the standard validation chain to verify the instance data’s conformance with the FDPG profiles. The pipeline’s output is a JSON file containing the validation results. For improved readability, we also generate a PDF report from these data.

For easy deployment at each site, we provide a configurable docker package containing all publicly available components via GitHub [24]. The pipeline does not require access to external networks and can be easily adapted for different profiles.

### FDPG Profiles and Underlying Assumptions

As previously established, the search ontology’s current representation and its FHIR profile representation are guided by making the most common and most harmonized instance data in Germany available and iteratively building on that for specific user groups. For the modulation of the current version, we assumed that hospitals across Germany, owing to the country’s billing system’s requirements, would consistently and correctly use *ICD-10-GM* [25], Operationen- und Prozedurenschlüssel (OPS), and Anatomical Therapeutic Chemical (ATC) codes for diagnosis, procedure, and medication, respectively. Consequently, these profiles can be easily expressed by mandating a coding from the respective code systems. The CDS profile for specimen already limits valid codings to the descendants of the SNOMED CT concept specimen. The search ontology reduces the valid codings further to a subset of the top 50 most common specimen codes. This information was already collected bottom-up before this analysis from metadata across sites, implying a high likelihood of minor to no discrepancies for Specimen in this study.

From a technical perspective, a specific coding is necessary to identify a criterion in the instance data. Beyond requiring a specific coding, the FDPG profiles commonly ensure the existence of a technical reference to the patient, which is crucial for the operation of the FHIR search. A single search ontology profile is sufficient for all modules except laboratory, following these guidelines. As previously established, the laboratory module presents a unique challenge due to its complexity and the interdependence of the value, the LOINC coding, and the number of codings.

Vreeman et al [26] identified that out of the 55,000 codes LOINC offers in practice, only a small subset is required to account for up to 99% of all laboratory observations [26]. Following their example, the MII established a “LOINC TOP300” subset that addresses 80% of all laboratory use cases in Germany [27]. The MIRACUM Metadata Repository (MDR) [11] makes this subset available and guides the current implementation in the feasibility tool of the FDPG.

The LOINC scale type associated with each concept [28] determines whether the laboratory result is quantitative or qualitative. While LOINC provides dimensional requirements, a wide set of Unified Code for Units of Measure (UCUM) units can fulfill that requirement. LOINC also provides exemplary units, but they are not mandatory. Fortunately, a UCUM representation is readily available for quantitative laboratory results in the MDR. The MDR, therefore, presents a machine-processable modulation of the interdependency of the LOINC and its quantitative value.

**Figure 5:**
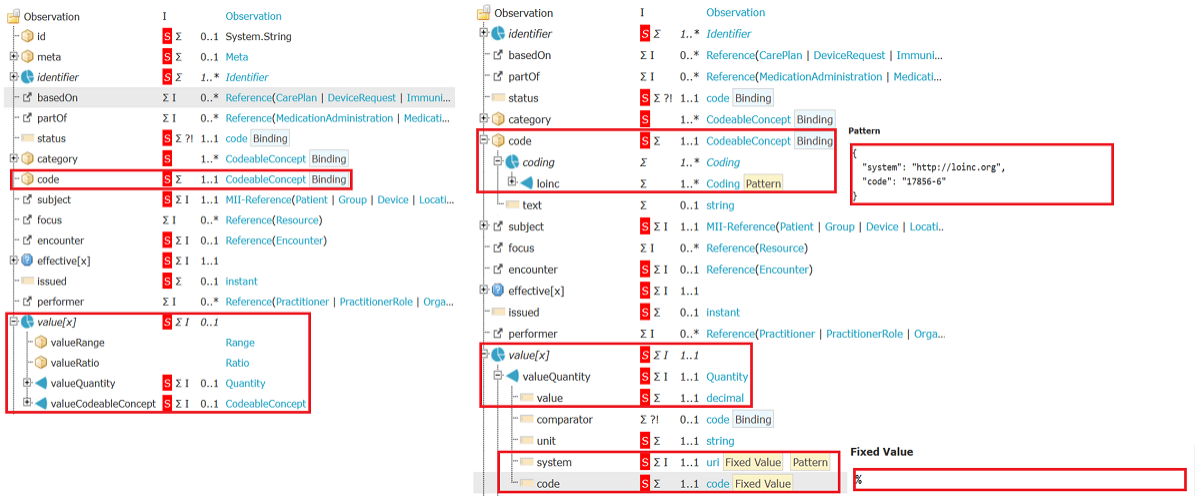
Core Data Set laboratory profile without interdependency between code and value (left) HbA1c profile with code value interdependency (right).

The MDR does not contain ValueSets for qualitative values, necessitating an alternative approach. If available, the LOINC answer list associated with the respective LOINC code is used for qualitative values, accessed via a terminology server. If unavailable, a general-purpose ValueSet defined by the MII is used. However, this ValueSet contains multiple representations of the same concept, such as 23 different representations for “Absence finding.” These various representations were reduced to a single value to enhance usability. Figure 5 illustrates how the information from the MDR is used to refine the CDS profile to specific FDPG profiles for each LOINC coding.

As the name suggests, the MDR is primarily used by the sites of this consortium and is not mandated for any sites in the context of the FDPG. Furthermore, given the nature of the laboratory profiles [29] within Germany concerning the representation of laboratory values cause expected variability.

The FDPG profiles are openly accessible on Simplifier [14].

Guided by these educated assumptions that led to the creation of the FDPG profiles, our hypothesis for this study is threefold. First, we anticipated high uniformity in applying *ICD-10-GM*, OPS, ATC, and specimen codes. Second, we hypothesized a considerably higher level of variability in the application and interpretation of LOINC codes, with regional disparities in the representation of laboratory values and their units playing a significant role. Third, we anticipated that our approach of refining the FDPG profiles based on the insight of the instance data would allow us to improve interoperability. This study aimed to probe these hypotheses, shedding light on the complexities of harmonizing and standardizing clinical data across different health care regions and coding systems in Germany.

### Ethical Considerations

This study did not require an ethics board’s approval as it did not involve analyzing individual patient data. Instead, summary statistics on data quality were generated at the participating respective sites, a practice in line with the applicable general data protection regulation.

## Results

### Overview

**Figure 6:**
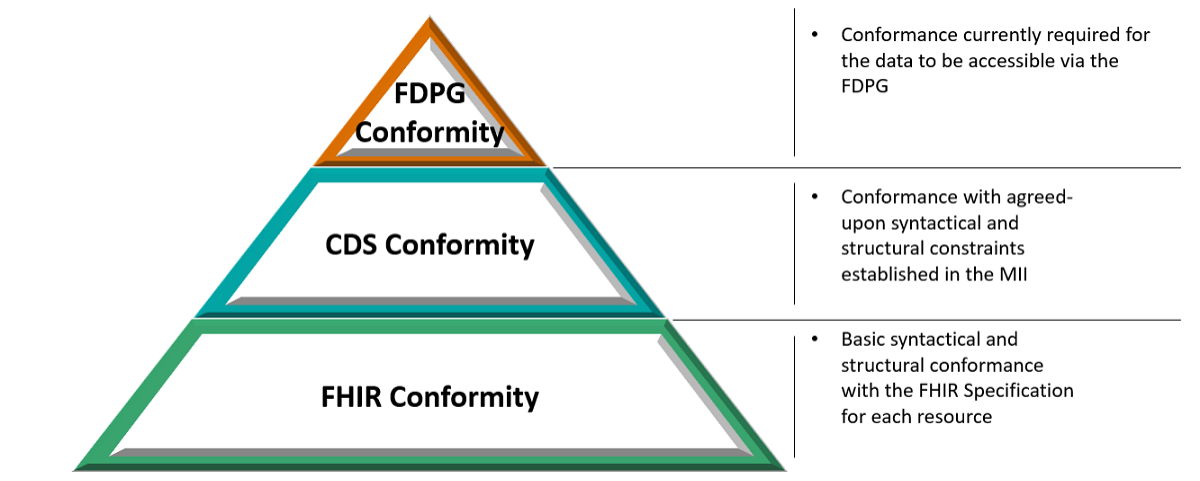
Data validation conformance levels. CDS: Core Data Set; FDPG: German Portal for Medical Research Data; FHIR: Fast Healthcare Interoperability Resources; MII: Medical Informatics Initiative.

To evaluate the methodology and tooling, we performed the discrepancy analysis over a wide range of sites (N=20) from all 4 consortia, including sites from western and eastern Germany and with different stages of DIC implementation. We differentiated 3 conformance levels when analyzing the results, as illustrated in Figure 6.

At a base level, all instance data must conform to the FHIR standard and the CDS. Deviations from these specifications are to be regarded as implementation errors. On the topmost level, the FDPG profiles are not binding for the DIC. Instead, they should be perceived as a description of the capabilities of the search ontology that allow insight into the subset of CDS conformant data currently searchable by the FDPG feasibility tool. Discrepancies on that level will be interpreted, and implications and possible solutions will be derived.

### CDS Compliance

Addressing CDS compliance, the surfaced discrepancies varied in nature and complexity, highlighting several key areas where adherence was lacking, spanning issues such as noncompliance with cardinalities, absent references, the nonfulfillment of rules stated in the profiles, and the use of incompatible codings. Notably, some of the key issues included the absence of the mandatory *status* attribute within the *MedicationAdministration* resources, which has a fundamental role in the context of the resource. Status codes that do not imply the completed administration are likely not relevant to clinical research, but if the status is not entered, no differentiation can be made.

A SNOMED CT–encoded *category* for *Procedure* was another prevalent discrepancy required when an OPS coding was used. While currently not the case, OPS codes might be used in a different context of a *Procedure* to differentiate in these cases the category that would be needed. Furthermore, errors in the *system* URL for codings caused validation errors, revealing an essential need for accuracy in defining codings.

While the above-listed findings in many cases do not directly affect the accessibility of the resources via the FDPG feasibility portal as these attributes are often not queried for, they emphasize the necessity for rigorous attention to CDS compliance in terms of detail and rigor in the data harmonization process for secondary use.

Beyond the issues that can be addressed with clear solutions, such as providing the required elements or adhering to specific rules or codings, we also found discrepancies that originate from misinterpretations of the implementation guides.

One example we found is using unit codes that do not stem from UCUM within the data element *MedicationAdministration.dosage.dose.code* in the Medication module. In discussing this discrepancy, we were pointed to the comment section for this element: “The preferred system is UCUM, but SNOMED CT can also be used (for customary units) or ISO 4217 for currency. The use context may also require a code from a particular system.” However, the profile requires “http://unitsofmeasure.org” as the system URL, causing a contradiction that should be addressed in the documentation.

### FDPG Compliance

As anticipated, the additional constraints in the FDPG profiles are the leading cause of discrepancies. However, many of our hypotheses are confirmed within this subset of instance data.

### Procedure, Condition, Medication, and Specimen

As expected, *procedure, condition, and medication resources are available with the codings* we anticipated. Unfortunately, our initial validation revealed that the terminology still caused discrepancies, but these can be traced back to the differences in the codes and the display. The national code systems used by the resources are currently not made available as FHIR resources but in their own format, leaving room for different interpretations and leading to different implementations to generate the displays. The Federal Institute for Drugs and Medical Devices (BfArM) responsible for the code systems is aware of this issue and has been assigned to create a central terminology service as the single source of truth for national code systems in Germany according to § 355 subsections 12 and 13 of the German Social Security Code V [30]. Our findings underline the importance of this intent. For the time being, we will ignore the display in the validation process.

Another discrepancy was using uppercase letters in OPS codes (5 of 20 sites) contrary to the FDPG feasibility portal’s expected lowercase codings for OPS. FHIR search to identify codings is case-sensitive unless the CodeSystem indicates case-insensitivity. FHIR server implementations to support case-insensitivity need to obtain this information from the code system information and adjust their internal search process accordingly. This feature is currently not implemented by the Blaze FHIR server. While the Blaze FHIR server might support this feature in the future, we tend not to make this assumption for all FHIR servers and, therefore, would advise adjusting the codings to have uniform upper or lower casing within one CodeSystem as a practical approach to this problem.

While the ATC code system is correctly coded, the possibility of representing medication information is another cause for discrepancies. The CDS defines 6 different representations for indicating that a medication has been prescribed to or taken by a patient. In FHIR, the prescription, the confirmed administration, and the statement of intake of drugs are modeled using different FHIR resources, MedicationRequest, MedicationAdministration, and MedicationStatement, respectively.

Furthermore, 2 options exist for the resources to link the specific code: the resources can reference a Medication resource or provide the CodeableConcept defining the medication. Currently, the FDPG feasibility portal only supports requesting MedicationAdministrations that reference a Medication resource. Moving forward, the FDPG feasibility tool must readdress requesting medication information.

The Specimen instance data could not be analyzed due to a software bug in the validation pipeline.

### Consent

For the Consent module, we encountered an erroneous understanding of the code system, setting the permissions given by the patient. As defined by the MII, the code system contains codes that provide specific permission and codes that imply a set of permissions. The implication for those implementing the Consent resource is the erroneous tendency to use only the most encompassing code in an assumed hierarchy, which currently is not explicitly modeled. It is necessary to list all relevant codes, even if their parent code is already present. Sites not including all subsequent codings do not negatively impact the data privacy but exclude patients that should be within the cohort when searching for more specific permission. In future versions, the currently implicit relationship might be modeled in the CodeSystem using the *part of* the relationship. Once changed, it will require the FDPG feasibility portal to request subsequent codes with their parent codes.

We also found consent information from the consent management software generic Informed Consent Service (gICS), widely adopted across the MII [31]. As the name implies, consent management software is used beyond the use case of federated feasibility queries in the MII. Therefore, consent resources made available by gICS are based on the more permissive profile defined by the official FHIR standard of the HL7 Germany Working Group Consent Management. These extended gICS consent resources can contain more attribute entries than consent resources based on the MII CDS Consent profile; for example, additional provision codings from the gICS defined code system for Consent resources that cause discrepancies when validated for our use case. Despite these findings, the consent resources remain compatible with our use case requirements, as they are based on the same standard, provided they contain all mandatory elements as defined by the MII CDS Consent profile.

### Observation (Laboratory Data)

The analysis of the *Observation* resource revealed findings that align with our hypotheses and highlight the intricacies of data harmonization. The inherent flexibility of the laboratory profile, the nonbinding nature of the MIRACUM MDR directives, and regional historical discrepancies manifested in significant heterogeneity.

A recurring issue is using qualitative values for quantitative LOINC codes and vice versa. We observed that the first case is more prominent than the latter, which is attributable to using codings for invalid measurements. In our federated feasibility use case, the indication of an invalid measurement bears a minimal consequence: the omission of patients with erroneous laboratory values would not adversely affect the validity of the result if sufficient patients are identified with a specific laboratory value. However, for use cases that evaluate the data, indicating a value’s invalidity is highly relevant and should be addressed in a standardized way. Solutions we uncovered range from using existing codes from various code systems with different granularity, such as indicating invalid measures using the SNOMED CT code for *invalid* or even a specific postcoordinated expression to provide additional insight about the cause, to using in-house code systems. The latter proves inadequate for achieving interoperability across various sites or when explaining the absence of data. In the future, clear guidance must be available to the sites to harmonize this information.

The leading cause for discrepancies that hinder the current accessibility of the laboratory values is the different UCUM units. However, the variety across the participating sites is not as wide as anticipated.

Within the instance data across the sites, we identified 368 different quantitative LOINC codes, with discrepancies in at least 1 site. Again, case sensitivity contributes to a significant number of discrepancies. Overall, 59 differences can be attributed to the inconsistencies between the use of the upper and lower letter “l” for liter. Overcoming this issue would lower the number of total discrepancies and the variety of different dimensions.

The BfArM defines a list of commonly used UCUM units [32]. According to our analysis against this list, discrepancies were attributed to units still predominantly used in eastern Germany. Some units, such as “Gpt/L” for giga particle per liter, are not included in the UCUM code system and are therefore unsupported. Another recurrent issue identified by this comparison is the use of the Greek letters (eg, “μ” instead of “u”). Considering only discrepancies caused by UCUM codes listed by the BfArM reduces the remaining differences to 248. This comparison with the prevalent UCUM units in Germany also revealed that 3 MDR entries differentiate from the BfArM list.

Moreover, 177 (71.4%) out of the 248 remaining discrepancies were due to different multiples of 10 in the representation (eg, ug/dl vs mg/L) with additional units that could be converted by applying more complex calculations such as converting mmol to g or mm [Hg] to kPa. Not having a medical background, we abstain from evaluating the correctness of the units, causing the remaining discrepancies. Importantly, we can show that there is a significant portion (298/368, 81%) of discrepancies that could be resolved by applying simple measures and, importantly, to lower variety in the discrepancies (66 out of the remaining 70 discrepancies would be caused by 1 different representation of a unit, and only 4 requiring the representation of the same value in 2 different units).

Multimedia Appendix 1 provides an excerpt of the table we created for this study and is one of the most important results of this study, as it can foster future developments. The LOINC codes most often found in the instance data are displayed.

## Discussion

### Principal Findings

Our work confirmed the need for sufficiently constrained profiles to inform the development of the federated feasibility tool. We presented tools and an approach that enables evidence-based decisions instead of the current educated guesses approach to create lower granularity CDS profiles. Performing the discrepancy analysis on the use case presented also granted significant insight into the data quality and alignment of the CDS among university hospital sites and the FDPG.

We also found first improvements in the data quality with the sites that addressed issues regarding the CDS identified in the data quality reports and subsequently undertook a reanalysis. This indicates that the presented tooling can improve data quality for agreed-upon profiles.

### Implications for the Future of the FDPG Feasibility Tool

Despite the success in other areas, the high number of discrepancies in laboratory values presents a unique challenge. From a product development perspective, considering the diverse nature of laboratory data, several options emerge:

- The feasibility tool could change its capabilities to query for the existence of a laboratory value. Insight into existing clinical studies from ClinicalTrials.gov indicates that researchers have a high demand for specifying specific ranges for laboratory values. Moreover, it could be argued that aligned with the General Data Protection Regulation, call for “data minimization” capabilities to further refinement even for feasibility queries could be deemed necessary.
- While theoretically feasible, expanding the range of selectable units to include all the existing ones would necessitate unit conversions by the researcher, likely negatively impacting usability and user awareness.
- Implementing on-the-fly conversions is typical in modern user interface design. However, given current technology constraints and the FDPG feasibility portal’s response time requirements, this approach is not viable for handling big data.

Under normal circumstances, this would indicate the necessity to pivot from the current implementation of the feasibility tool; fortunately, the collaborative nature of the MII offers an alternative solution:

- Ideally, alignment of the laboratory values would stem from the primary source system; our empirical findings show (as portrayed in the *Results* section) that for the TOP300 LOINC Codes, the discrepancies can be overcome by applying rather simplistic measures to improve data harmonization. The data already being in a standardized format further enable the development of 1 tooling that suits all sites’ needs. The MIRACUM consortium has initiated preliminary efforts in this direction and is available to all MII members [33]. While we would highly advise against mapping LOINC Codings, as implemented in the tooling, it showcases the feasibility of providing a conversion tool. We acknowledge that such tooling might require significant quality assurance (the tool LUMA [34] can support ensuring the used units match the dimensions defined in LOINC) or even the approval as software as a medical device given the existing expertise in the MII [35] and the reoccurring demand for further data harmonization for, for example, distributed machine learning, we regard the endeavor worthwhile.

Harmonizing the data would also give users a broader range of units by converting the selected value to the harmonized representation. There still might be cases where data harmonization of the instance data is not feasible. A fallback to the previously discussed solutions would still prevail in these cases.

Once the main issue of aligning the representation of the values in the search ontology is overcome, it will be necessary to expand the list of LOINC codings and their interdependencies continuously. Already, we found that 15 of the 20 sites had additional LOINC codings in the relatively small sample size of 500 Observations.

### Related Work

**Figure 7:**
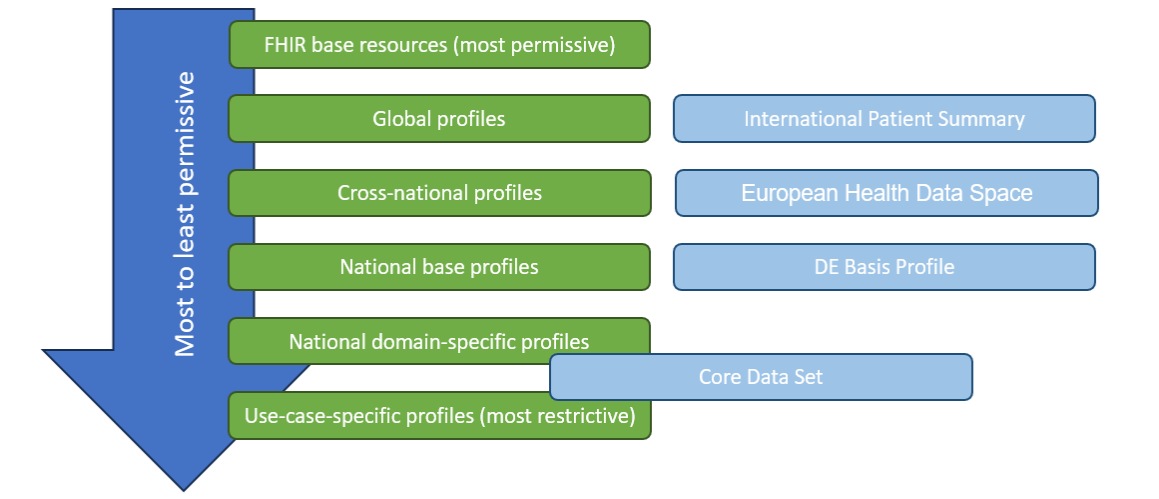
Layered Fast Healthcare Interoperability Resources profile model. DE: Deutsche (German).

Addressing the different granularities of FHIR resources and international, national, and domain-specific profiles is a topic that has seen more traction in recent years. With the International Patient Summary (International Organization for Standardization 27269) defining a minimal baseline of elements that need to be present in the electronic health record of a patient and its implementation in FHIR profiles to address the use case of “unplanned, cross border care,” it is likely to see a rise of interdependent FHIR profiles. For European countries specifically, the goal of a shared European Health Data Space [36] will require defining an additional layer between the international and national levels. These profiles are not intended to serve all use cases. Figure 7 outlines the different layers based on the work of Vreeman (Vreeman, DJ, unpublished data, July 2023) and Aassve [37] and the role of existing implementation guides within these layers. The layered approach from minimally restrictive to sufficiently restrictive data modeling also offers the opportunity to promote reuse. The role of the CDS is not clearly defined within this model, while the FDPG profiles are sufficiently restrictive for the use case of federated search.

Kramer [38] pinpointed a discernible gap in reusability within FHIR, as revealed through their scrutiny of 125 implementation guides. The layered approach enables the definition of reusable extensions, promotes terminology use, and provides clear guidance on the existing profiles that can be enacted as a baseline.

While some may advocate for an all-encompassing top-down approach for particularly restrictive profiles, we believe that the existing instance data will inevitably require aligning for different reasons: existing instance data based on a less-stringent layer, missing restrictive profiles, or insufficient insight on real-world instance data when creating the profiles. Once this alignment is achieved, the established tooling in this work can be further used for quality assessment. While our work goes beyond the data quality assessment (DQA) of the CDS, it is inherently a part of our examination, if only regarding conformance.

Draeger et al [39] and Kamdje-Wabo et al [40] performed a DQA across DICs in the MII using an R script to analyze specific elements in the instance data regarding their conformance, completeness, and plausibility as defined by Kahn et al [41]. Their findings align with our findings regarding conformance and completeness on a smaller sample set but go much more in depth when assessing plausibility. Ideally, future assessments will synthesize both methodologies: harnessing the robustness of FHIR profile validations as a foundation and superimposing intricate plausibility evaluations. Within the context of the MII, the MIRACUM DQA tool [42-44] assesses the data quality based on abstract data set definitions in the consortium-provided MIRACUM MDR. The same MDR underpinned our FHIR profile formulation for laboratory values.

Consequently, the sites that used the MIRACUM DQA tool had fewer discrepancies in our analysis. Still in the context of the MII but beyond the FHIR standard, the work of Tute et al [45] also applied rule-based evaluation on specific data elements using R. However, the data source they analyzed was an openEHR repository queried with AQL statements.

In the broader scheme of data quality of medical data, we also find the reoccurring topic of addressing data ambiguity. Schmidt et al [46] delve into the significance of data quality in observational health research and the different quality indicators, presenting a comprehensive framework that not only highlights the challenges but also offers software solutions in R to tackle them.

In conclusion, while the push toward defining and refining FHIR profiles and embracing standardized data formats is a significant step forward, it remains a piece of a giant puzzle to achieve interoperability and high data quality in the medical domain, which will ultimately require a multifaceted approach.

### Limitations

The presented approach requires high effort, limiting its inherent iterative nature. Therefore, it is essential to use all information obtained in each cycle to make informed decisions on the maximally constrained profiles that ultimately lead to profiles that the sites can use independently to access their data quality. The high effort is also why the initial run of discrepancy analysis is already based on educated assumptions. Notably, the method works even if only the required cardinalities and arbitrary values for the required attributes are specified in the profiles. Consequently, the first pass would cause maximum discrepancies and full insight into the instance data.

With the high alignment of educated assumptions, the analysis only evaluates data that should already match the search ontology criteria. LOINC Codes outside the TOP300, different codings for medications, diagnosis, or procedures are not part of the performed analysis and must be revisited once made available in the search ontology.

Compared with data analysis scripts, an advantage of this approach is its easy adaptation and reuse of the validation pipeline to perform DQAs for completeness and correctness. However, while aspects of plausibility can be found in our results, these are not directly related to our approach and must be addressed separately.

Finally, we did not analyze the *Patient* resource due to the federated nature of our analysis and privacy concerns. Being the central part of every feasibility query, sites should use the existing tooling to ensure conformance.

### Outlook

We created an individual feedback PDF report containing all discrepancies for every participating site based on our findings. We hope that this feedback will allow the sites to address the identified discrepancies that are not caused by the FDPG profiles but rather are contradictions between the CDS and their ETL processes.

After reaching sufficient maturity, the FDPG profiles will be used to update the search ontology.

Further iterations, when expanding the ontology and between time periods, of the presented approach could be summed up in an iteration study providing insight into the continuous development of the central platform and the DIC.

This work revealed the necessity of extending collaborations between the CDS team, FDPG team, and DICs. While many discussions are still open at this point, first adjustments are already being made, that is, by the representatives of the CDS Consent module who have already implemented our recommendation of using the “part-of” relationship in the CodeSystems hierarchy. Now, it falls to the FDPG team to adjust the feasibility query accordingly in a future release.

We believe that it is pivotal to build on the presented approach to provide sites with tooling that enables them to verify their data quality concerning the CDS and identify if their instance data are available via the FDPG feasibility tool, allowing them to adjust their data or demand adjustments of the feasibility tooling if discrepancies arise. While the current tooling was tailored for this study, further adjustments can and should be made to provide sites with a more actionable report; that is, the report should provide a working link to a resource where a validation error occurred, allowing on-site users to have full access to all information.

We see a high demand for the presented approach. Whether partly to ensure data quality or fully to refine top-down defined profiles with evidence-based information to enhance interoperability. We provide a generic approach and sufficient tooling to make it usable for use cases beyond the FDPG, requiring only slight adjustments to work with other profiles.

## Appendix

Multimedia Appendix 1 Analysis of LOINC (Logical Observation Identifiers Names and Codes) code units and derivations across multiple clinical sites and the German Portal for Medical Research Data.

## Abbreviations

ATC: Anatomical Therapeutic Chemical
BfArM: Federal Institute for Drugs and Medical Devices
CDS: Core Data Set
DIC: data integration center
DQA: data quality assessment
ETL: extract, transfer, load
FDPG: German Portal for Medical Research Data
FHIR: Fast Healthcare Interoperability Resources
gICS: generic Informed Consent Service
HL7: Health Level 7
*ICD-10-GM*: *German Modification of the International Statistical Classification of Diseases and Related Health Problems, 10th Revision*
LOINC: Logical Observation Identifiers Names and Codes
MDR: Metadata Repository
MII: Medical Informatics Initiative
MIRACUM: Medical Informatics in Research and Care in University Medicine
OPS: Operationen- und Prozedurenschlüssel
SNOMED CT: Systematized Nomenclature of Medicine—Clinical Terms
UCUM: Unified Code for Units of Measure
